# Illegal Harvesting within a Protected Area: Spatial Distribution of Activities, Social Drivers of Wild Meat Consumption, and Wildlife Conservation

**DOI:** 10.3390/ani13050771

**Published:** 2023-02-21

**Authors:** Sarah Bortolamiol, Thierry Feuillet, Wilson Kagoro, Rukia Namirembe, Edward Asalu, Sabrina Krief

**Affiliations:** 1 CNRS–UMR 7533 Laboratoire Dynamiques Sociales et Recomposition des Espaces (LADYSS), Campus Condorcet, 5 Cours des Humanités, 93322 Aubervilliers, France; 2 Sebitoli Chimpanzee Project (SCP), Great Apes Conservation Project (GACP), Sebitoli Station, Kibale National Park, Fort Portal, Uganda; 3 Department of Geography, Université de Caen Normandie, CNRS–UMR 6266 Identité et Différenciation de l’Espace, de l’Environnement et des Sociétés (IDEES), Campus 1, Esplanade de la Paix, 14032 Caen, France; 4 Uganda Wildlife Authority (UWA), Plot 7 Kira Road, Kampala P.O. Box 3530, Uganda; 5 Rwenzori Commodities Ltd., Buzirasagama Tea Factory & Estates, Fort Portal P.O. Box 167, Uganda; 6 Eco-Anthropologie (EA), Musée de l’Homme, Muséum National d’Histoire Naturelle, CNRS, Université de Paris, 17 Place du Trocadéro, 75116 Paris, France

**Keywords:** biodiversity management, wild meat, chimpanzee, social science, illegal harvesting, spatial analysis, Uganda, wildlife conservation

## Abstract

**Simple Summary:**

Wild meat is a primary source of protein for many rural populations and its illegal harvest can threaten worldwide wildlife conservation efforts. Near tropical forests, wild meat can be an alternative to domestic meat consumption for different reasons (economy, access, culture, etc.). We aimed to understand drivers of illegal activities affecting wildlife in a hotspot of biodiversity (Sebitoli, Kibale National Park, Uganda) hosting threatened species (including chimpanzees) and bordered by agricultural landscapes with high human densities. Illegal activities were mapped, and individual interviews were conducted. We highlighted that being a young man coming from districts contiguous to Kibale National Park particularly raises the odds of consuming wild meat. This study might help to identify and recommend sustainable social and environmental alternatives to manage biodiversity.

**Abstract:**

The African tropical forests host an inestimable number of resources, including food, medicine, vegetal and animal species. Among them, chimpanzees are threatened with extinction by human activities affecting their habitats, such as forest product harvesting, and/or more directly, snaring and trafficking. We aimed to better understand the spatial distribution of these illegal activities, and the reasons for setting snares and consuming wild meat in an agricultural landscape (subsistence farming and cash crops) densely populated near a protected area (Sebitoli, Northern part of Kibale National Park, Uganda). To carry out this study, we combined GPS records of illegal activities collected with group counts (in total, *n* = 339 tea workers, 678 villagers, and 1885 children) and individual interviews (*n* = 74 tea workers, 42 villagers, and 35 children). A quarter of illegal activities collected (*n* = 1661) targeted animal resources and about 60% were recorded in specific areas (southwest and northeast) of the Sebitoli chimpanzee home range. Wild meat consumption, which is illegal in Uganda, is a relatively common practice among participants (17.1% to 54.1% of respondents depending on actor types and census methods). However, consumers declared that they eat wild meat unfrequently (0.6 to 2.8 times per year). Being a young man coming from districts contiguous to Kibale National Park particularly raises the odds of consuming wild meat. Such an analysis contributes to the understanding of wild meat hunting among traditional rural and agricultural societies from East Africa.

## 1. Introduction

Besides high human, developmental, and financial investments in wildlife conservation worldwide [1,2,3] and access limitation to protected areas (PAs) [4], illegal wild meat hunting (here, defined as poaching) continues to threaten terrestrial mammals’ extinction [5]. Wild meat (a wild animal killed for consumption, which is different from bushmeat killed for trade [6]) serves as a vital resource in many rural, lower-income regions of sub-Saharan Africa, and near tropical forests it is often a primary source of protein for rural populations [7]. Its consumption tends to prevail in areas with greater biodiversity indices, which also frequently experience higher poverty and food insecurity [8,9]. Nevertheless, the dependence of households on wild meat is particularly lacking documentation in East Africa [10].

Recent increases worldwide in illegal wildlife harvesting are less related to local use and traditions in provenance countries [11] than to an increase in wealth and a decrease in resources in countries importing harvested species or body parts (sent from Africa to Asia; [12]). Therefore, wild meat hunting and poaching (hunting becomes poaching when the practice does not follow the law/rules) can respond to different motivations such as harvesting wild meat for consumption or trade (locally and abroad [13]). To regulate both practices, most countries require hunters to have hunting permits and refer to quotas. Following international guidelines, countries also regulate subsistence hunting through laws that restrict hunting certain species (protected or non-protected), during certain time periods (months, day/night, seasons, etc.), and using specific weapons/tools (riffles, snares, traps, dogs, etc.). However, in practice, it is not this simple: reforming and adapting the regulatory framework of local communities hunting for food is necessary [14], conservation militarization is questionable [15], and development projects aiming to reduce wild meat hunting and/or poaching and maintain food security need a large, adaptable, and clear framework [16,17,18].

More research on the prevalence of wild meat consumption and its drivers has been conducted in West Africa and Central Africa than in East Africa. Additionally, across Africa, cultural, sociopsychological, and sociodemographical factors driving wild meat consumers’ behaviors remain understudied [19,20,21]. Some studies show that wildlife illegal harvesting is determined by non-exhaustive ecological, social, and economic drivers such as the frequency of game species, poverty, countries/areas of provenance, ethnic groups, cultural values/beliefs, revenge from crop-raiding animals, lack of access to alternative incomes, heath issues, the distance to local markets, and/or the frequency of patrols [10,22,23,24,25,26,27,28,29,30]. Species abundance and wild meat affordability are often cited as main predictors of harvest levels [31,32], variables such as taste or health issues are often considered secondary factors for wild meat food choices [33], and remoteness or landscape characteristics have been rarely investigated [34]. Indeed, the driving factors of wild meat consumption are complex and variable [16]. In the end, motives to consume legal or illegal wild meat are distinct, mainly relying on case-by-case studies [35], and are more likely to result from a combination of factors, with some being more prevalent than others depending on each case. Consequently, different responses should target different factors [36], which is our aim here. In East Africa, and specifically, in Uganda, wild meat hunting is forbidden within parks and their surrounding areas [37]. The continued existence of a global market for trade in natural resources acquired through illegal means, as well as a lack of data and research for decision and policy makers to implement successful wildlife management and regulation, has been noticed [38]. Between 2005 and 2009, over 71% of households interviewed on their incomes (cash or subsistence) that were located around two protected areas and one unprotected area reported having participated in wild meat hunting [39]. Such practices were related to different reasons in different areas [28,40,41]. Yet, conservation strategies used over the last two to five decades in/around a Ugandan National Park seem to have been effective for protecting the park and animals living within [41].

Here, we aim to add a new wild meat consumption case in East Africa to the literature, and identify the drivers of wild meat consumption around a PA in order to design better local alternatives and interventions [16,42,43] to reduce pressure on wildlife in a context where both human density and animal species diversity are high. We focus on the Sebitoli area, located in the extreme north of Kibale National Park (hereafter, Kibale NP), southwestern Uganda. Besides a combination of international and national policies trying to conserve different wildlife forms [37,44], the illegal activity index is high in the area [41]. Chimpanzees (*Pan troglodytes schweinfurthii*; CITES and IUCN) are endangered. In the Sebitoli community they are indirectly victims of illegal snare injuries [45]. Indeed, snares are likely directed at small game meat (duikers, bush pigs, etc.) not at primates. However, it threatens primate conservation and a non-negligible part of tourism income [46]. Additionally, over the course of the study a chimpanzee was found dead and smoked in a surrounding household (august 2016). Therefore, to estimate the threat animals face in the Sebitoli area, we aimed to obtain a spatial understanding of illegal activities targeting wildlife (objective 1), to identify the targeted species and evaluate the frequency of wild meat consumption (objective 2), and to discuss, in general, drivers of domestic and wild meat consumption in the area (objective 3) with six-year-old data. This study will contribute to the PA’s management plan, help identify and compare wild meat consumption drivers with those identified in other studies, and add to the understanding of this practice in Uganda in particular, and in East Africa in general.

## 2. Materials and Methods

### 2.1. Study Area and Inhabitants

Kibale NP (795 km^2^) was established in 1993 and is currently under Uganda Wildlife Authority (UWA) management. It covers three districts: Kyenjojo, Kabarole, and Kamwenge. Local communities can access the park by request to the UWA through resource use agreements [47]. The area of Sebitoli (25 km^2^) is densely populated with humans and wildlife [48,49,50]. Landscapes are anthropogenic, combining tea, plantain, and eucalyptus plantations; small-scale food gardens; a busy tarmac road [51]; and a high human population density that tripled between 1959 and 1990 and is currently as high as 293 inhabitants/km^2^ in subcounties including Kibale NP [52]. As wild animals from the park (especially elephants, baboons, and chimpanzees) raid crops in local communities’ gardens (maize, cassava, bananas, etc.), human–wildlife conflicts around Kibale NP are recurrent [53,54].

Batooro and Bakiga are the main tribes in the area [48], and Batooro are particularly represented in the north of the park [52]. Batooro are rare meat eaters and their feeding taboos regarding meat consumption can favor the conservation of some wild species, including primates [55]. Additionally, there is not a large wild meat market around Kibale NP: most demands are minimal because most illegally harvested meat (bush pigs and small antelopes) are consumed locally and are not supplied to large external markets [56].

Outside of the park, inhabitants practice subsistence farming, and some families work in the large tea plantations [50]. Migrant workers from other districts (not contiguous to Kibale NP) or other countries such as Rwanda can comprise up to 40–60% of the tea workforce, depending on the size of the tea concessions [57]. Tea plantations cover a particularly large surface outside of the park and play a buffering role between the park’s edge and palatable crops (maize, potatoes, millet, etc.). However, few studies have focused on tea workers’ way of life [58].

### 2.2. Snare Removal and Illegal Activity Patrols in the Sebitoli Area

The Sebitoli area benefits from the presence of UWA rangers patrolling the park to monitor illegal activities and interacting with the local communities neighboring the park about wildlife conservation. It also has benefitted from the presence of the Sebitoli Chimpanzee Project (SCP) since 2008.

In addition to UWA patrols, research teams studying chimpanzees in Kibale NP also run snare removal projects (Kanyawara Snare Removal Project; Ngogo Chimpanzee Project; and SCP). At SCP, three local community members were recruited for this task beginning on May 29th, 2015 (study period: 29 May 2015–30 November 2016, 271 patrol days). Using transects and their knowledge of the park, they patrolled the Sebitoli chimpanzee home range 5/7 days for 6–8 h/day using GPS to record illegal activity locations where the targeting of natural resources occurred (snaring, tree cutting, charcoal burning, etc.) and datasheets to record illegal activity characteristics (date, hour, GPS location, type and oldness of activity, amount of evidence, etc.). They disactivated and confiscated any evidence (snares, spears, cables, etc.) that they brought back to the Sebitoli research site, where they were stored. This spatial information was later mapped by SB.

### 2.3. Individual Interview and Group Count

We aimed to compare the legal and illegal animal protein access of tea workers and villagers since both populations live and work within a close distance of the chimpanzee home range and seem to experience sociodemographic, economic, and cultural differences [58]. Children were also included in our sampling, as they represent an important proportion of the local populations [59] and are sensitive to environmental degradation, such as species loss [60].

Group counts and individual interviews were carried by the first author (SB) and a local translator. The two methods were designed to be complementary to estimate wild meat consumption in the area. In group counts, votes (via boxes and hands) were opportunistically set up before other SCP activities and described wild meat consumption in a quantitative way from a large sample of respondents (no sociodemographic characteristics identified outside of the actor type—villager, tea worker, and children). In semi-structured interviews, wild meat consumption was qualified with more details and time from a smaller sample of respondents (including sociodemographic characteristics and the discourse of participants).

#### 2.3.1. Individual Interviews

##### Interview Process

**Figure A1:**
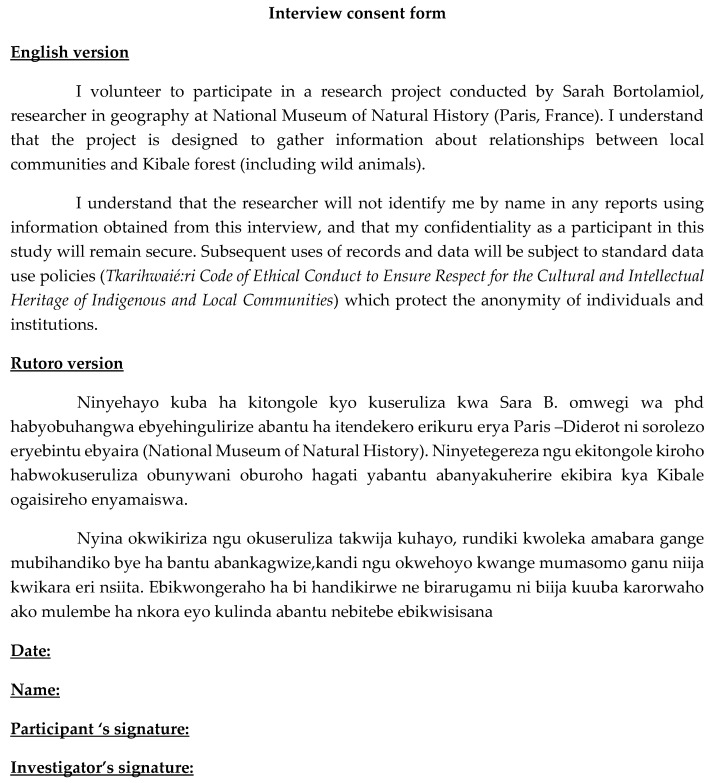
Interview consent form used around Sebitoli area, Kibale National Park, Uganda. Interview respondent and interviewer each had a copy.

**Figure A2:**
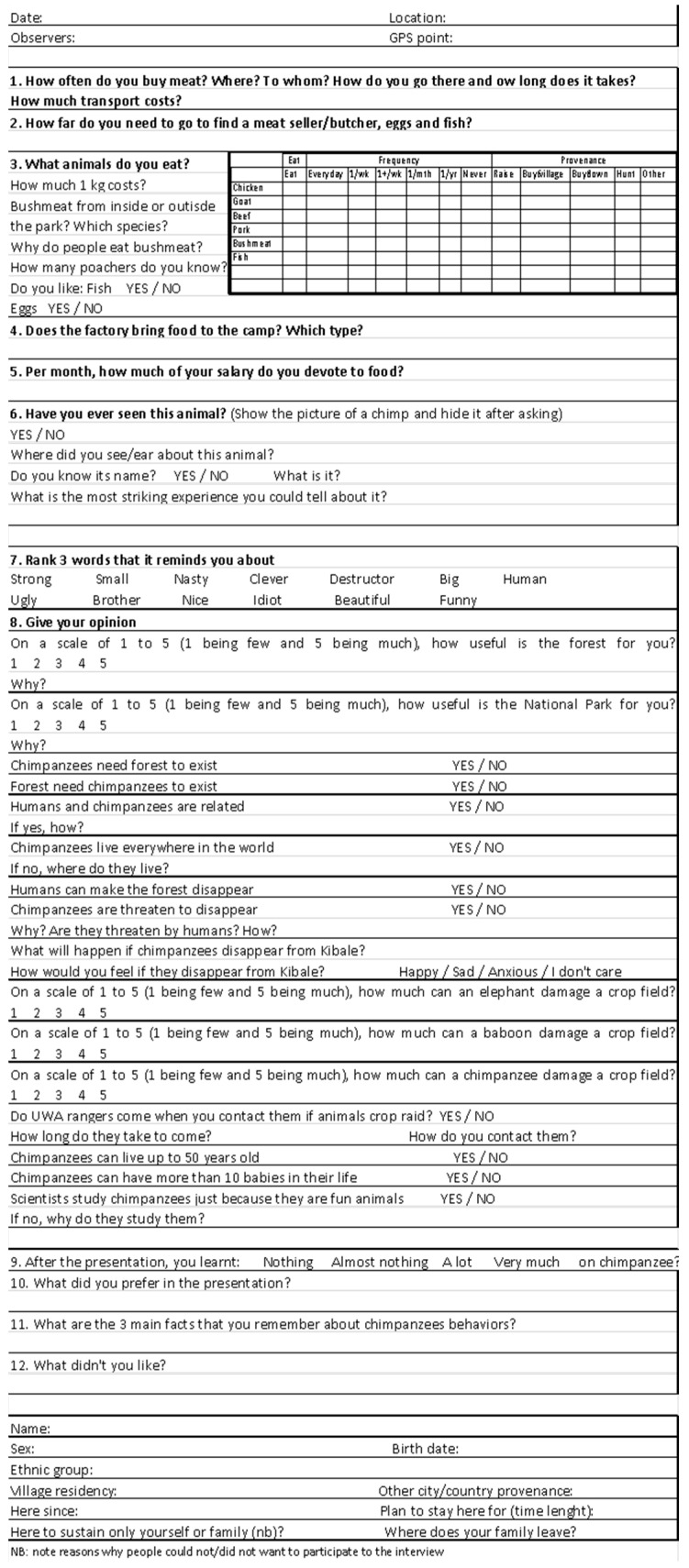
Interview form used around Sebitoli area, Kibale National Park, Uganda.

Tea workers, villagers, and children had the context of the survey verbally explained to them (e.g., a postdoctoral research study on domestic and wild meat consumption, and relationships with wildlife), the anonymity of their name and the possibility to withdraw from the survey at any time were guaranteed, and both SB and interviewees signed a consent form (Figure A1) after each participant agreed to participate in the survey. A 30 min semi-structured interview was then administered by SB and a translator (*n* total interviewees = 151; see Figure A2 for detailed questions). Questions focused on: (1) domestic animal protein consumption (frequency, buying location, and transport means); (2) wild meat consumption and illegal activities knowledge (frequency of consuming wild meat, and if respondents did not eat bushmeat they were asked the frequency they come across with it, number of poachers known, targeted species, and reasons to eat wild meat); (3) relationship with wildlife (chimpanzee knowledge and crop-raiding levels); and (4) sociodemographic characteristics. Most questions were closed, but questions related to wild meat consumption were more open and allowed for free speech. In this case, responses were later classified by SB into the main categories.

##### Tea Workers

A tea company granted us access to tea worker camps and allowed us to carry out interviews with their employees during working hours between May and July 2016. SB came to an agreement with 8 tea estate managers on when to carry out interviews at tea camps located around the Sebitoli chimpanzee home range (average distance of 309 m, range: 151–576 m from Kibale NP). In the morning, interviewers came to a tea camp, presented the survey to tea workers, and interviewed workers who volunteered one after the other in an isolated place near the tea fields. A total of 74 workers were interviewed (4 to 11 workers interviewed per camp).

The number of tea workers working at each camp varied between 62–178 workers (median: 143). Some workers reside in villages, whereas some reside at the tea camps for free, with a proportion varying between estates (18.6–88.7%; median: 49.6%) (Rwenzori Commodities Ltd., Fort Portal, Uganda, unpublished data). At the tea camps, workers benefit from individual bedrooms where they can occasionally host their family and share common cooking fireplaces and sanitary installations with other workers staying at the camp. Additionally, the tea company provides free lunches to tea workers.

##### Villagers and Children

Four village chiefs (LC1) and two head teachers (primary schools) located around the Sebitoli chimpanzee home range granted us permission to interview local inhabitants and children, choosing days when they would be available between October and November 2016. The same voluntary selection process was applied as for the tea workers. A total of 42 adults (10–12 per village) living around Kibale NP (average distance of 300 m, range: 2–572 m from the park’s edge) were interviewed at their household in four villages during their daily activities. Additionally, 35 children (15–20 per school) living at the park’s edge were also interviewed in two schools (average distance of 2000 m, range: 1349–2668 m from Kibale NP) during class hours.

#### 2.3.2. Group Counts

We took advantage of a chimpanzee awareness presentation on chimpanzee biology and behavior conducted by SCP that is a regular program of the project to ask questions about wild meat consumption. The program was conducted in 11 tea worker camps, 20 villages, and 14 schools located around the Sebitoli chimpanzee home range between June and December 2016. More than 3900 persons attended presentations, mainly in schools (*n* = 2722 persons, median: 181 pers/school, range: 22–598; 1 nursery school, 8 primary schools, 1 high school, and 4 institutes/vocational schools), villages (*n* = 739 persons, median: 49 pers/village, range: 15–99) and tea worker camps (*n* = 456 persons, median: 41 pers/camp, range: 25–64).

**Figure 1:**
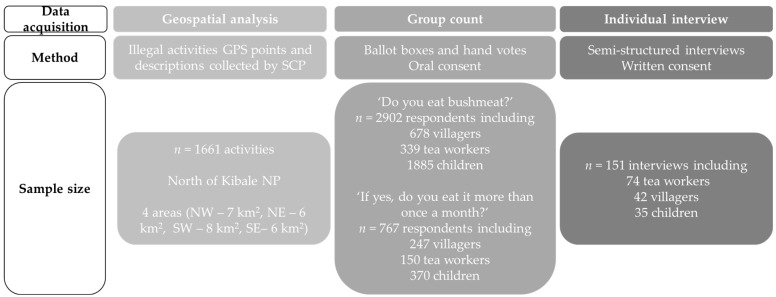
Data acquisition types, collection methods, and sample sizes used in determining spatial distribution of illegal activities and wild meat consumption analyses in Sebitoli area, Kibale National Park, Uganda.

**Figure A3:**
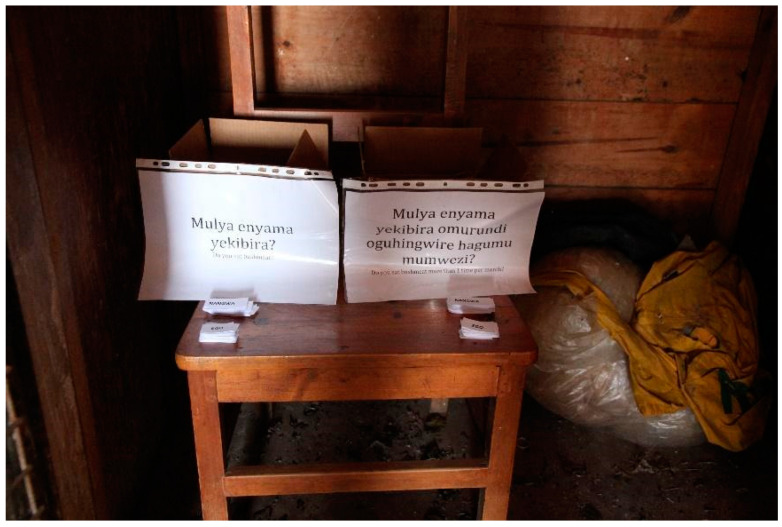
Ballot boxes used for group counts in one tea camp to answer the questions: “Do you eat bushmeat?”, and if yes, “Do you eat it more than once a month?” around Sebitoli area, Kibale National Park, Uganda.

No specific sampling method was used to select respondents. After setting up an appropriate time with tea managers, LC1s, and head teachers, the aim of the presentation was explained to the people who came, and it was explained that on a voluntary and anonymous basis, the SCP team would like to ask them two questions about wild meat consumption. Informed consent was verbally given by adults and children, and only people who volunteered to participate contributed. A total of 2902 persons (Figure 1) answered: “Do you eat bushmeat?”, and if yes, “Do you eat bushmeat more than once a month?”. Two different methods to collect responses were used for the different age groups. The ballot-box method was used with adults (villagers and tea workers): two boxes were set out of sight and participants dropped a paper (one paper yes, and one paper no) in each of these boxes to answer questions (Figure A3). This method has the advantage of reducing the social desirability bias [61] and the disadvantage of showing a trend without the possibility to link individual behavior and explanatory variables [62]. With children in schools, hand votes were used to respond to the same two questions. This method has the advantage of reducing children’s confusion that can occur with two sets of boxes, but has a social desirability bias due to other pupils’ presence (teachers were asked to leave the classroom) during votes.

### 2.4. Analyses

**Figure A4:**
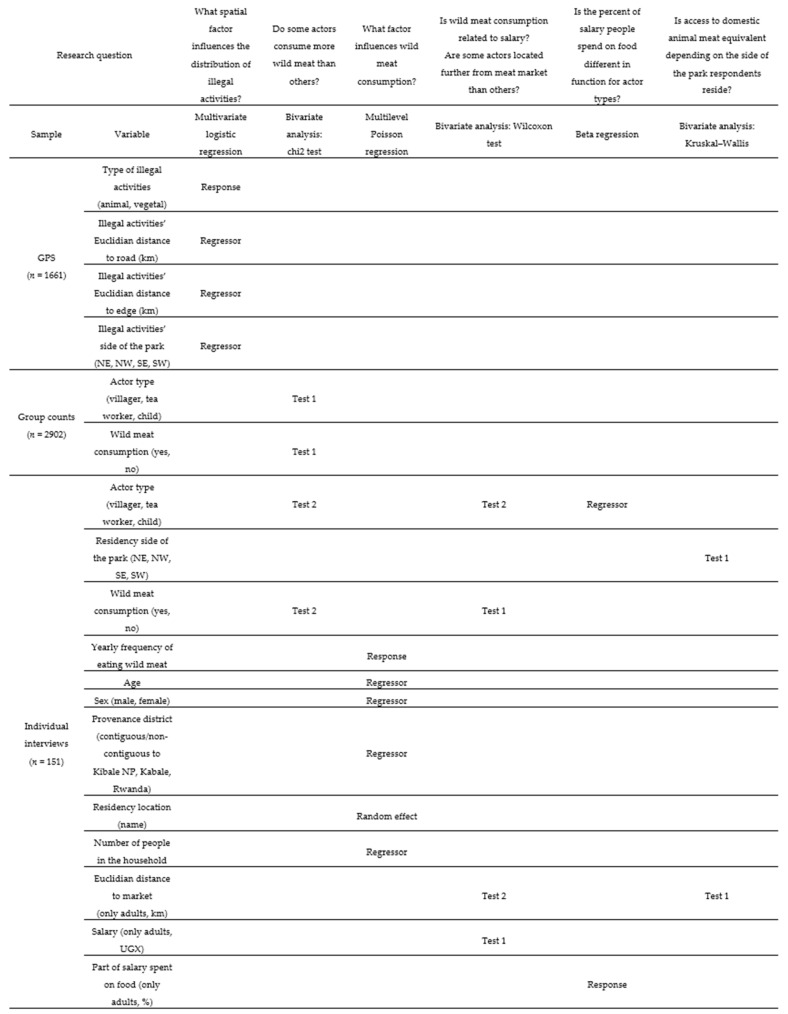
Variables used for illegal activity and wild meat consumption statistical modeling of key questions, Sebitoli area, Kibale National Park, Uganda.

Data acquisition types, methods, sample sizes, research questions, and analyses are presented in Figure 1 and Figure A4 to help synthesize the key information.

#### 2.4.1. Geospatial Analyses

Illegal activity locations were recorded by the SCP snare removal team during their patrol. SB took the locations of each tea camp, village, and school and georeferenced them all using GPS coordinates (GPS Garmin 64s; ArcGIS 10.2; geodesic system–WGS 84; cartographic projection–UTM 36 N). To facilitate spatial analysis, the Sebitoli chimpanzee home range was divided in four areas of relative equivalent size (northwest (NW) 7 km^2^, northeast (NE) 6 km^2^, southwest (SW) 8 km^2^, and southeast (SE) 6 km^2^). Euclidean distances of illegal activities to the edge and to the road were assessed to evaluate which border of the forest was more at risk. The Euclidean distance was also calculated between participants’ residences and the sellers of domestic animal protein by using the centroids of villages/trading centers/camps to evaluate their accessibility.

#### 2.4.2. Statistical Analyses

We used bi-/multivariate analyses and regression models to estimate the relationships between variables depending on key research questions (Figure A4).

**Figure A5:**
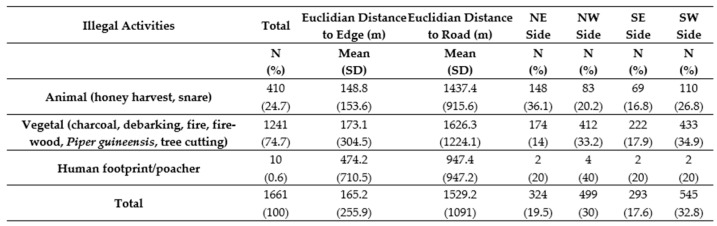
Number of illegal activities collected by SCP (29/05/2015–30/11/2016) per section of Sebitoli area, Kibale National Park, Uganda.

With the GPS sample (*n* = 1661), the relationships between types of illegal activities, Euclidean distances to the road and the edge, and the sides of the park (Figure A5) were assessed though a multiple logistic regression. In the group count sample (*n* = 2902), we used a chi^2^ test to estimate the relationship between actor type and wild meat consumption (Test 1, Figure A4). In the individual interview sample (*n* = 151), five distinct analyses were conducted:

(i) A chi^2^ test was used to estimate the relationship between actor type and wild meat consumption (Test 2, Figure A4).

(ii) To assess which variables were associated with wild meat consumption, we carried out a multiple generalized linear regression model. Given the distribution of the response variable (frequency of consuming wild meat, i.e., a count variable) and the hierarchical structure of the data (151 individuals nested into 28 residency locations), we estimated the following random intercept multilevel (i.e., mixed) Poisson model:

$$y_{ij}\sim \mathrm{Poisson}\;\left(\mu_{ij}\right)\;\mathrm{for}\;i=1,\ldots ,151\mathrm{and}j=1,\ldots ,28\;\ln\left(\mu_{ij}\right)=\beta_{0}+\beta_{1}x_{i1}+\cdots +\beta_{k}x_{ik}+(e_{ij}+\mu_{0j})$$

where *i* denotes the individuals, *j* is the residency locations, *β* is the parameters estimated by the maximum likelihood (Laplace approximation), *x_ik_* is the value of the *k^th^* covariate for individual *i,* and *e_ij_* and *u_oj_* are the two residual components at both the individual and location residency levels. Adding location residency as a random effect allowed us to account for between-location heterogeneity while controlling for within-location spatial dependence. Multicollinearity among regressors was previously verified through VIF values. Covariates with a VIF > 2 were removed, leading to the exclusion of the side of the park and ethnicity variables. The final model includes the five following regressors: sex, age, actor type (children, tea workers, or villagers), provenance (contiguous/non-contiguous to Kibale NP, Kabale, and Rwanda) and number of people in the household.

(iii) We used the Wilcoxon test to assess the relationship between wild meat consumption and salary (Test 1, Figure A4), as well as for Euclidean distance to market and actor type (Test 2, Figure A4).

(iv) The relationship between the percent of salary respondents spent on food (response variable) and actor type was estimated through a beta regression. Beta regression is a class of model used when the response variable is beta-distributed, which is commonly true when it is between 0 and 1 for such proportions. As other generalized linear models, beta regression relates the mean response to the regressors through a link function.

(v) To assess whether the Euclidean distance to the market was equivalent in function of the side of the park respondents reside in, we used a Kruskal–Wallis test.

In all the GLM presented in this study, coefficients were exponentiated (i.e., leading to odds ratios (OR)) for an easier interpretation of elasticities. R software [63] with betareg and lme4 packages [64,65] was used to perform statistical analyses.

## 3. Results

### 3.1. Illegal Activity Spatial Distribution

**Figure 2:**
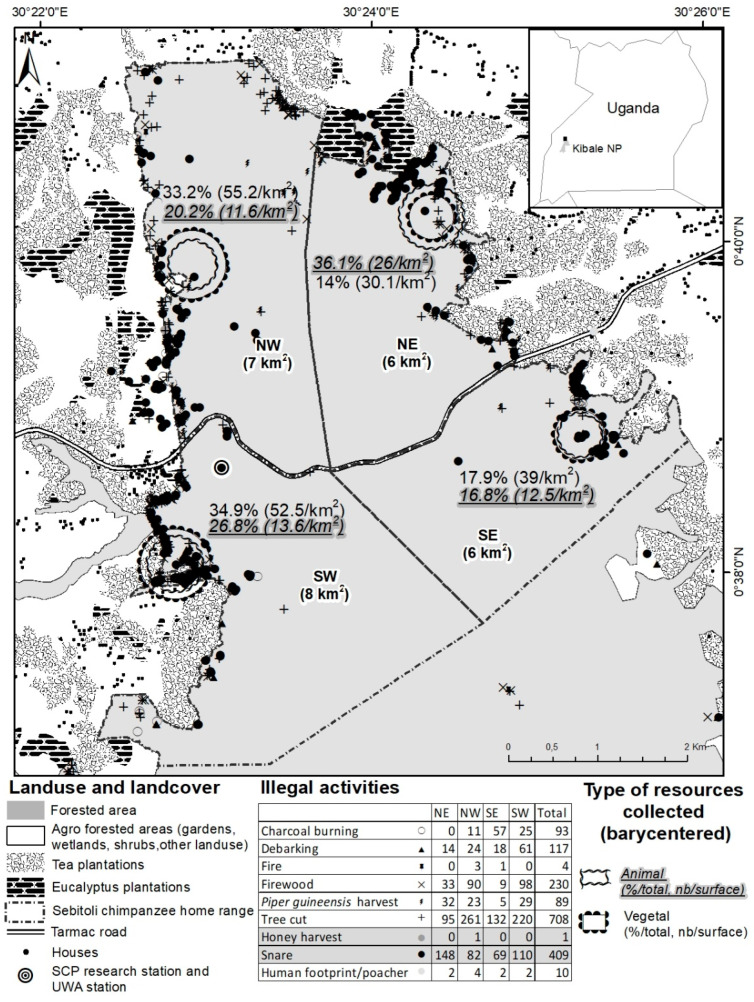
Illegal activities identified by SCP snare removal team (29 May 2015–30 November 2016), Sebitoli area, Kibale National Park, Uganda.

**Figure A6:**
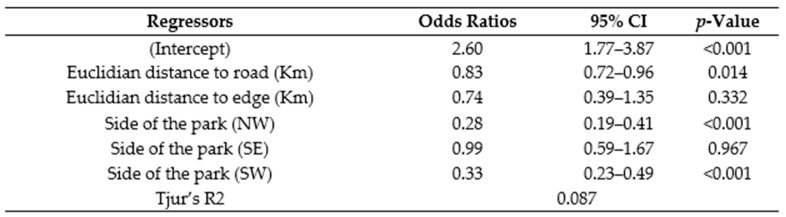
Multiple logistic regression between type of illegal activities, Euclidian distances to the road and the edge, and sides of Sebitoli area, Kibale National Park.

Between May 2015 and November 2016, the SCP georeferenced a total of 1661 illegal activities (9 different types, targeting animal and vegetal species) inside (*n* = 1436) and outside (*n* = 255) the park in the Sebitoli area (Figure 2). Overall, illegal activities targeted more vegetal (74.7%) than animal resources (24.7% including 24.6% of snares), and were found closer to the park’s edge (mean: 165.2 m) than the road (mean: 1529.2 m) (Figure A5). Animal resources were more likely to be found in the NE (concentration of 1/3 of snares collected in the area during the study period). On that side, the tea company had eight tea camps/factories at the border of the forest. The logistic regression confirmed that animal resources have fewer chances to be found in the northwestern (OR = 0.28, 95% CI = [0.19–0.41], *p* < 0.001) and southern (OR = 0.33, 95% CI = [0.23–0.49], *p* < 0.001) sides of the park compared to the NE side and the area close to the road (OR = 0.83, 95% CI = [0.72–0.96], *p* < 0.05; Figure A6).

### 3.2. Wild Meat Consumption in Group Counts

Wild meat was said to be eaten by 27.3% of overall participants according to the group counts (*n* = 2902, Table 1). It was reported to be more consumed among villagers (47.9%) than tea workers (31%) or children (19.3%), and this difference was significant (*χ*^2^ = 209.08, df = 2, *p* < 0.001). Additionally, the proportion of children eating wild meat more regularly throughout the year was more important (38.9%) compared to villagers (31.2%) or tea workers (26%).

### 3.3. Wild Meat Consumption in Individual Interviews

#### 3.3.1. Descriptive Statistics

**Figure A7:**
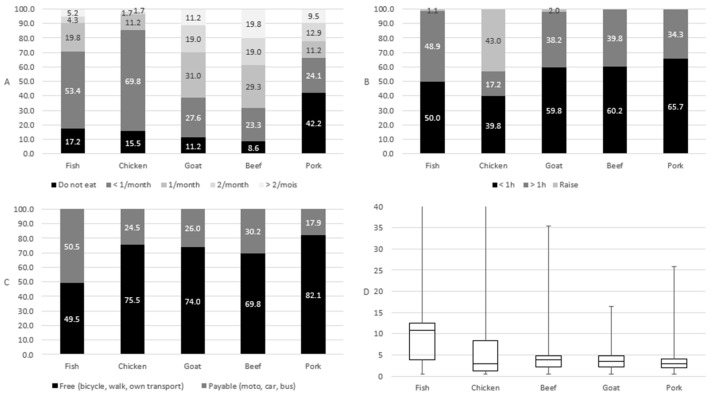
Frequency of domestic animal protein consumption (**A**); access time to domestic animal protein among consumers who eat, buy, and raise domestic animals (**B**); access mean to domestic animal protein among consumers who eat and buy domestic animals (**C**); Euclidian distance (kilometer) to domestic animal protein among consumers who eat and buy domestic animals (**D**) around Sebitoli area, Kibale National Park, Uganda.

According to individual interviews, meat consumption is generally low in the Sebitoli area. About 60% of respondents do not eat domestic animal protein (fish, chicken, goat, beef, and pork; Figure A7), eat it less than once a month, or eat it once a month when it is accessible (reduced time, and diversity of locations and choices; Figure A7). Additionally, even if about 40% of respondents in individual interviews declare to eat wild meat, it happens rarely: 2.8 times/year for tea workers, 1.3 times/year for villagers, and 0.6 times/year for children (Table 2).

Individual interview results differ from group counts. Here, more tea workers declare to eat wild meat (54.1%) compared to villagers (35.7%) and children (17.1%), and this difference is significant (*χ*^2^ = 13.975, df = 2, *p* < 0.001). From the interviews, the main reasons to eat wild meat were that its cheaper in price compared to domestic meat (43.7%), unknown motives (20.5%), culture/tradition (19.9%), taste (9.9%), and other motives (medicine, distance to access domestic meat, and revenge against crop-raiding animals–6%). Participants in interviews mainly consumed bushbuck (24.5%; *Tragelaphus scriptus*), red duiker (15.9%; *Cephalophus harveyi*), bush pig (12.6%; *Potamochoerus porcus*), and edible rat (6.6%; unknown species). Primates were cited by 5.3% of respondents (seven out of eight were children). Additionally, 26.5% of respondents did not know which species were consumed.

#### 3.3.2. Results of the Mixed Poisson Model of Wild Meat Consumption Frequency

The mixed Poisson model (location residencies as a random variable) exhibited four significant variables (Table 3). Among them, the strongest association was sex. On average, males consume 3.08 (95% CI = [1.83–5.19]) times more wild meat than females, whereas all other variables held constant. Respondents coming from the Kabale district consume 0.50 (95% CI = 0.29–0.85) less than those coming from districts contiguous to Kibale NP. Regarding continuous variables, age was negatively associated with wild meat consumption (IRR = 0.96, 95% CI = [0.94–0.99]; meaning that a one-year increase in age was associated with 0.04% less meat consumption), whereas the number of people sustained in the household had a positive effect (IRR = 1.13, 95% CI = [1.03–1.23]). The regression’s interclass correlation coefficient (ICC = 0.43) indicates a strong effect of residency locations in the model, but no clear patterns appear between tea camps and village residencies. In summary, being a young man coming from districts contiguous to Kibale NP particularly raises the odds of consuming wild meat when all other covariates are kept constant and controlled for within-residency location dependence.

#### 3.3.3. Complementary Factors from Individual Interviews (Adults Only)

This section offers complementary data analyses from variables that were not available for the entire individual interview sample. Indeed, for some questions (salary and market location for domestic meat) only adults were able to answer. Yet, this information is useful when discussing our results.

##### Revenues and Percentage Spent on Food

The difference between adult tea workers’ (*n* = 74) and villagers’ (*n* = 42) revenues were not significant (Wilcoxon test W = 1496, *p* = 0.75; Figure A4). Villagers tend to spend more money on food than tea workers (97.6% vs. 13.5% had a self-sufficient garden and most tea workers lived in camps without their family where they were offered free lunches) while their main occupation was subsistence agriculture (61.9%), but this relationship was not significant according to the beta regression model (Figure A4).

##### Geographical Distance to Domestic Meat

**Figure A8:**
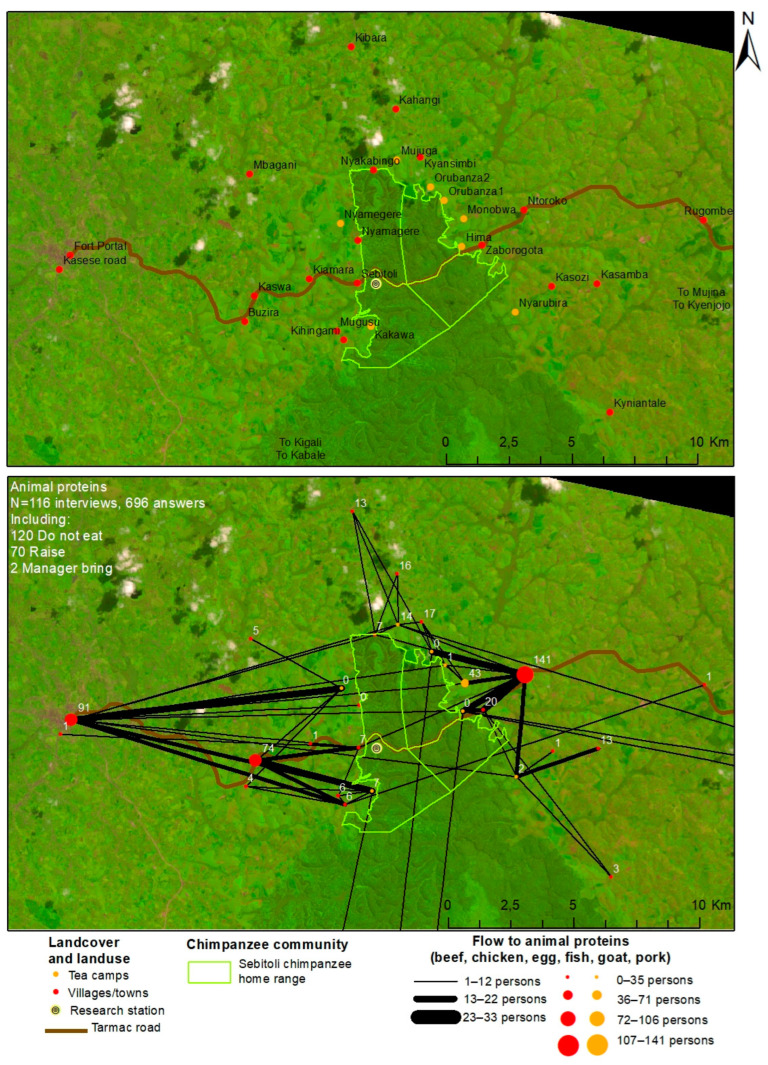
Residency locations of domestic animal meat buyers and locations where they acquire all types of meat combined in Sebitoli area, Kibale National Park, Uganda (May–December 2016).

**Figure A9:**
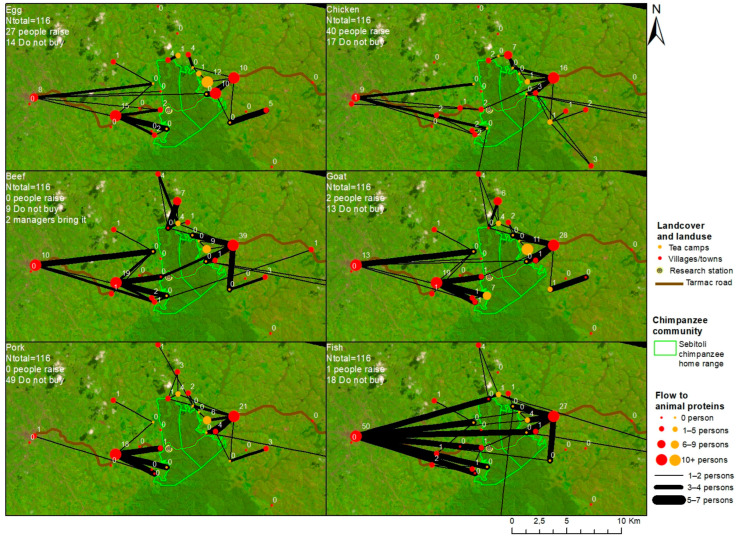
Residency locations of domestic animal meat buyers and locations where they acquire each type of meat in Sebitoli area, Kibale National Park, Uganda (May–December 2016).

Three market centers, Fort Portal, Kaswa, and Ntoroko, with distances of 16 km, 4.4 km, and 2.5 km from the forest edge, respectively, attracted domestic meat buyers (Figure A8 and Figure A9). However, to access domestic animal protein, adult respondents travelled a longer or shorter distance. Significant differences existed in the distance to access domestic meat yearly (Kruskal–Wallis chi-squared test= 26.434, df = 3, *p* < 0.001), and this difference was stronger between the NE and SW (respective mean: 25.6 km and 32.8 km, pairwise comparison *p* < 0.001) than the NE and SE (respective mean: 25.6 km and 20 km, *p* < 0.01), and the SE and SW (*p* < 0.05). No significant difference existed between the NW (mean: 24.5 km) and other areas of Sebitoli. Additionally, a significant difference existed between tea workers’ and adult villagers’ Euclidean distance to access/buy domestic meat (Wilcoxon test *W* = 1191.5, *p* < 0.05; villager mean: 21.5 km; tea worker mean: 29.2 km).

## 4. Discussion

Over an 18-month study period, 24.6% of overall censused illegal activities consisted of targeting animals (snares), putting them at risk to be trapped, especially in the northeastern and southwestern sides and at the forest edges. Results estimating wild meat consumption among the three groups differed between group count and individual interview methods. Children were always consuming wild meat the least, but different results regarding adult and tea worker consumption occurred depending on the methods used. Results from the individual interview method may be more reliable as more time and attention were taken in collecting information.

### 4.1. Spatial-, Social-, and Species-Related Factors Affecting Wild Meat Consumption

In the Sebitoli area, the park is probably accessed from its edges (rather than the road) to extract resources. This is common in such a conservation context [41,66,67], especially for forest products [41,68,69]. Surprisingly, the setting of snares occurred close to the forest edge, where chances of being caught are likely higher. More snares were collected in the NE (Figure 1) where crop-raiding impacts are of a medium level relative to other locations around the park [53], but they were also collected in the SW of the Sebitoli area where group count participants mostly declared to eat wild meat, distances are longer to access domestic meat, and crop-raiding impacts are high [53]. This edge effect supports the belief that large PAs provide a good option to prevent species losses [70,71]. The stability of illegal activity hotspots (e.g., location in previous years) may be a good predictor for illegal activities in PAs [72], which can be helpful to establish de-snaring strategies [73].

Our interview methods highlight that between 17.1% and 54.1% of our samples declared to eat wild meat in the Sebitoli area, but more on an occasional (a few times a year) than regular basis. These proportions are lower than the 71% respondents from the three Ugandan sites [39], and are close, on average, to the 31% of households wanting to access wild meat around Kibale NP [53]. Regarding our results, we reviewed common but non-exhaustive motives found in previous research that should be considered if trying to increase the sustainability of wild meat consumption at a study site:

- Price: In interviews, wild meat appearing to be cheaper than domestic meat (or free if the harvester) is a common pattern among poor African households that can explain its consumption frequency compared to other meats [26,74,75]. As in other studies, our results highlight that respondents’ frequency of eating wild meat increases with the number of people in the household [20,76], as eating wild meat can decrease spending on food [77], and it is an important factor in poverty reduction in rural areas [22,78,79,80].
- Taste: Species preferred as wild meat, such as bushbuck, red duiker and wild pig, are known to be hunted for wild meat in Kibale [81]. None of them are threatened with extinction [82]. Wild meat is believed to be “sweeter” than livestock meat and several people believe that it contains fewer chemicals than other meat around the park [83]. Across Africa, wild meat is generally preferred to domestic meat, with arguments of higher quality and better taste [26,84]. However, sometimes, the wild meat sold is misrepresented as another species of wild meat [85]. Ungulates constitute more than the majority of all hunted animals in West, Central, and East Africa [27,31,86], and among wild meat, antelopes are often cited as a preferred species [10]. Compared to other meats, ungulates have a superior quantity of meat with less fat [26,87], and a greater amount of edible protein per unit of live weight than domestic animals (Ledger, 1967, cited in [26].) Bush pigs are also advantageous animal protein sources, representing an important quantity of meat with a low level of total fat [88].
- Remoteness of domestic meat: The northeastern part of the Sebitoli area experiences more snares targeting wildlife and it is also where the distance to access domestic meat is one of the longest. Most studies and field programs on wild meat consumption focus on individual preferences and/or the role of sociodemographic variables in such behavior. Remoteness and, more generally, landscape characteristics, which can limit access to marketed domestic animal protein, are less frequently used to account for wild meat consumption despite it being a relevant factor linked to dependence on hunting for subsistence [34,89,90,91].

### 4.2. Increasing Human Livelihoods to Promote Wildlife Conservation

In the Kabarole district (connected to Kibale NP), animal protein contributes a small percentage of the total protein intake, as families seem to strive to eat and buy fish or red meat once a week and chicken (the most expensive animal protein) is eaten more rarely [92]. The Batooro and Bakiga are mainly subsistence-level agriculturalists [93], and have many taboos regarding meat; it is considered “pure” when it is coming from ruminants (or, generally, animals eating plants), but “impure” and inconsumable when coming from carnivore and omnivore animals [55].

Relative to other foods, meat and fish have a high “diet impact ratio’’ (e.g., a high environmental impact per calorie of food supplied [94]) and the amount of meat consumed through the world is not predicted to decrease [95]. Therefore, increasing domestic animal production is not necessarily the most suitable perspective for biodiversity conservation [18,96,97,98]. In Uganda, increasing the supply of domestic animal protein and reducing its price locally could be a viable policy option to reduce wild meat quantity demand and hunting pressure on Kibale NP’s wildlife, as it was also suggested in other locations [18]. As for tea workers specifically, implementing lunches provided by the tea company with meat (for example, once a week) may be an incentive for wild meat consumption as well. Therefore, changes in diet would have to be followed up carefully as some studies suggest that a substitution away from wild meat can mean a significant increase in fish consumption [18,79], which could also cause biodiversity conservation issues.

New ways to involve and sustain local communities’ needs while contributing to wildlife conservation are developing. For example, mini/micro-livestock (the rearing of small wild mammals [99]), eating insects [100], or sun-dried meat in times of food shortage [101] can enhance the animal product supply in sub-Saharan Africa. Regarding the taste issues mentioned earlier, meat should be produced locally to be positively perceived [19], and it could be sold in the Sebitoli area through community markets for conservation [102]. Other means, such as reducing the impact of crop raiding with the use of wildlife deterrents to avoid risks of reprisals from agriculturalists [40,57,73,92], incentivizing poachers [103,104], and developing programs about wildlife conservation and/or spiritual associated knowledge may be effective [20,105,106,107,108,109,110] at strengthening and combining the positive support and sustainable attitudes toward wildlife.

## 5. Conclusions

A combination of factors leads to illegal hunting and consumption of wildlife in the Sebitoli area. The driving factors are spatial (proximity to park’s edges) and social (sex, age, provenance, and household size). The wild meat hunting crisis is a fundamentally distressing problem to address because it is intimately tied to human development challenges such as food insecurity, emergent disease risks, and land-use changes [111]. Therefore, efforts to promote biodiversity conservation need to be integrated, conjoint, multilevel, interdisciplinary, progressive, and sustained for the long term.

## Figures and Tables

**Table 1:** Wild meat consumption among group count participants, Sebitoli area, Kibale National Park, Uganda.

	Consume Wild Meat			More than Once a Month (12 Times a Year)		
	Total	Yes	No	Total	Yes	No
Group counts	*n*	*n*	*n*	*n*	*n*	*n*
	%	%	%	%	%	%
Villagers	678	325	353	247	77	170
	23.3	47.9	52.1	32.2	31.2	68.8
Tea workers	339	105	234	150	39	111
	11.7	31	69	19.6	26	74
Children	1885	363	1522	370	144	226
	65	19.3	80.7	48.2	38.9	61.1
Total	2902	793	2109	767	260	507
	100	27.3	72.7	100	33.9	66.1

**Table 2:** Interview respondents’ description, Sebitoli area, Kibale National Park, Uganda.

Variables	Modalities	Interviews of Tea Camps (*n* = 74)	Interviews of Villagers (*n* = 42)	Interviews of Children (*n* = 35)
Wild meat consumption (percent)	Yes	54.1	35.7	17.1
	No	45.9	64.3	82.9
Frequency of consuming wild meat (count)	Mean (SD) Range	2.8 (12.3)	1.3 (3.9)	0.6 (2.1)
		0–104	0–24	0–12
Sex (percent)	Woman	21.6	47.6	54.3
	Man	78.4	52.4	45.7
Age (year)	Mean (SD)	29.4 (6.7)	37.4 (14.7)	11.7 (2)
	Range	18–50	18–78	7–16
Provenance (percent)	District contiguous to Kibale NP	39.2	71.4	88.5
	Kabale district	32.4	16.7	2.9
	District non-contiguous to Kibale NP	8.1	7.1	8.6
	Rwanda	20.3	4.8	0
People sustained (Number)	Mean (SD) Range	4.4 (2.2)	6.1 (3.1)	6.2 (2.3)
		1–15	1–14	2–11

**Table 3:** Mixed Poisson model of wild meat consumption frequency in Sebitoli area, Kibale National Park, Uganda.

Fixed Effects			
Regressors	Incidence Rate Ratios	95% CI	*p*-Value
(Intercept)	0.44	0.15–1.29	0.133
Actor Type (Tea worker)	1.61	0.90–2.88	0.17
Actor Type (Child)	0.44	0.16–1.16	0.096
Sex (Male)	3.08	1.83–5.19	<0.001
Provenance (Kabale)	0.50	0.29–0.85	0.011
Provenance (Noncontiguous)	0.43	0.18–1.05	0.065
Provenance (Rwanda)	0.81	0.44–1.50	0.56
Age	0.96	0.94–0.99	0.002
Number of people in household	1.13	1.03–1.23	0.009
Random effects			
σ2	0.97		
τ00 village	0.74		
ICC	0.43		
N villages	28		
Observations	151		
Conditional R2	0.560		

## Data Availability

The data presented here are sensitive (related to illegal practices). They are only available on request and under certain conditions to respect the authors’ commitment to participants.
